# Altered Primary Motor Cortex Neuronal Activity in a Rat Model of Harmaline-Induced Tremor During Thalamic Deep Brain Stimulation

**DOI:** 10.3389/fncel.2019.00448

**Published:** 2019-10-15

**Authors:** Jihyun Lee, Su-youne Chang

**Affiliations:** ^1^Laboratory of Brain & Cognitive Sciences for Convergence Medicine, College of Medicine, Hallym University, Anyang, South Korea; ^2^Department of Neurologic Surgery, Department of Physiology and Biomedical Engineering, Mayo Clinic, Rochester, MN, United States

**Keywords:** essential tremor, harmaline, deep brain stimulation, primary motor cortex, thalamus

## Abstract

Although deep brain stimulation (DBS) is a clinically effective surgical treatment for essential tremor (ET), and its neurophysiological mechanisms are not fully understood. As the motor thalamus is the most popular DBS target for ET, and it is known that the thalamic nucleus plays a key role in relaying information about the external environment to the cerebral cortex, it is important to investigate mechanisms of thalamic DBS in the context of the cerebello-thalamo-cortical neuronal network. To examine this, we measured single-unit neuronal activities in the resting state in M1 during VL thalamic DBS in harmaline-induced tremor rats and analyzed neuronal activity patterns in the thalamo-cortical circuit. Four activity patterns – including oscillatory burst, oscillatory non-burst, irregular burst, and irregular non-burst – were identified by harmaline administration; and those firing patterns were differentially affected by VL thalamic DBS, which seems to drive pathologic cortical signals to signals in normal status. As specific neuronal firing patterns like oscillation or burst are considered important for information processing, our results suggest that VL thalamic DBS may modify pathophysiologic relay information rather than simply inhibit the information transmission.

## Introduction

Essential tremor (ET) is a neurological condition that causes shaking or rhythmic motion in body parts. ET is characterized by a positional and/or volitional 4- to 12-Hz tremor that can affect all body parts (Marshall, 1962) and affects nearly 1% of the world’s population, increasing to 4% of those over age 40 (Müller et al., 2016). Although the preferred treatment for ET is pharmacology based, medications for ET may decrease in efficacy over time or not be efficacious (Chopra et al., 2013). It is estimated that pharmacological treatment can improve tremor in only 50% of patients (Lyons et al., 2003). Therefore, deep brain stimulation (DBS) becomes an alternative treatment option when medication is not effective or not tolerated in patients with disabling tremor (Zhang et al., 2010; Crowell and Shah, 2016).

Ventral intermediate nucleus of the thalamus (VIM) is a well-established DBS target for ET (Deuschl and Bergman, 2002), because the VIM is a cerebellar relay nucleus in the ventrolateral (VL) part of the thalamus and is known to be involved in ET (Benabid et al., 1991; Hua et al., 1998; Schuurman et al., 2000). The VIM shows abnormal neuronal discharges in humans with ET (Deuschl and Bergman, 2002). The cerebral cortex is also a part of the tremor-generating neuronal network (Hellwig et al., 2001; Raethjen et al., 2007). The corticothalamic anatomical connections are well established (Raethjen et al., 2007; Raethjen and Deuschl, 2012). Thalamic neurons receive robust input from corticothalamic feedback neurons, thereby allowing the cortex to communicate continuously with the thalamus during sensory processing (Briggs and Usrey, 2008). Corticothalamic feedback neurons reside in cortical layer 6 and give rise to axons that terminate both in the thalamus and in the layers of the cortex receiving thalamic input (Sherman, 2005; Wearden et al., 2006). Recently, several studies have suggested that cortical thickness could be used as an ET diagnosis with a high accuracy (Chung et al., 2013; Cerasa et al., 2014; Serrano et al., 2017; Benito-León et al., 2019), which may partly reflect a decrease in the amount of corticothalamic connections. In the study of connectivity for the cerebello-thalamo-cortical network in humans, when tremor is severed, M1 has reduced cerebellar functional connectivity, whereas the thalamus has increased cerebellar functional connectivity (Lenka et al., 2017). Thus, there is evidence for alterations in cerebello-thalamo-cortical circuit activities directly influencing ET.

In the neurophysiological view, rhythmic firing patterns such as oscillations and bursts have been considered as a way to process information in the brain (Varela et al., 2001; Izhikevich et al., 2003). Therefore, we hypothesized that DBS at the VL thalamus (VLT), which is a homolog of VIM in humans, may act through changing neuronal firing patterns in the primary motor cortex (M1). To test our hypothesis, we investigated changes of M1 neuronal activities while high-frequency VL thalamic DBS (130 Hz) was applied in harmaline-induced tremor rats. Here, we demonstrated that four neuronal discharge patterns exist in M1 and VLT and those were changed by harmaline. Our data also show that high-frequency thalamic DBS changed the proportion of four different firing patterns of M1 activities; and these changes have a residual effect, which was maintained even after the termination of DBS in harmaline-administered rats.

## Materials and Methods

### Animals

Subjects were adult Sprague–Dawley rats weighing 270–350 g (34 rats for behavior only, 8 rats for M1 recording, 15 rats for VLT recording). Animals were maintained under standard laboratory conditions on a 12/12-h light/dark cycle (lights on at 7:00 a.m.), and they had free access to food and water. All experimental procedures were approved by the Mayo Clinic Institutional Animal Care and Use Committee for experimental animals.

### Tremor Induction and Measurement

Tremor was induced by intraperitoneal (i.p.) injection of harmaline (harmaline HCL, Sigma-Aldrich, St. Louis, MO, United States). Harmaline was dissolved in saline and administered at a dose volume of 5, 7.5, 10, and 20 mg/kg. Tremor was measured with the force-plate actimeter (FPA, BASi, Lawrence, KS). A force-sensing actimeter was confined to a horizontal sensing area by an enclosure suspended a short distance (2 mm) above the upper surface of the force plate (440 mm^2^ × 440 mm^2^) (Fowler et al., 2001). Four force transducers existing below the corners of the plate recorded the animals’ position on a Cartesian plane and measured the force exerted on the plate at each time point. Data were collected and stored in the duration of 10.24 s as 1 frame with 100 points per second as a sampling frequency. Rats were placed on the force plate, and their movements were measured for 4 h 16 min (1,500 frames). Harmaline was injected following 20 min of movement recording for a baseline. The experiment was performed over five consecutive days.

### DBS Electrode Implantation Surgery and Application

Rats were anesthetized with 2–3% isofluorane and mounted in a stereotactic device (David Kopf Instruments, Tujunga, CA, United States). Two screws were located on the skull for attaching the electrode. Customized concentric bipolar electrodes were 1.07 mm in the diameter of the outer shell, and the inner and outer cores were made with stainless steel wires, 300 and 700 μm in diameter, respectively. The electrode was stereotactically implanted into the VLT [anterior–posterior (AP) – 2.3 mm, medial–lateral (ML) ± 1.8 mm from the bregma, and dorsal – ventral (DV) – 5.6 mm from the brain surface, according to the Paxinos and Watson (1998) rat brain atlas. The electrode was attached to the skull with screws using dental cement. After implantation surgery, the animals were placed in a cage for at least 1 week to recuperate.

After full recovery from the electrode implantation surgery, the animals were placed in a force-plate actometer. The electrode was connected to a stimulus generator (AM system, World Precision Instruments, Sarasota, FL, United States). After 20 min of baseline movement recording, high-frequency stimulation (130 Hz, rectangular pulse width 100 μs) was applied for 10 min followed by 10 min off stimulation. After that, low-frequency stimulation (10 Hz, rectangular pulse width 10 μs) was applied for 10 min followed by 10 min off stimulation. Then, harmaline was administered intraperitoneally at a dose of 10 mg/kg; and 30 min later, the stimulation procedure described above was repeated.

### Tremor Analysis

Tremor data were filtered by the Hanning window and converted to power spectra of frequency by a fast Fourier transform (FFT) on each frame of the experiment using MATLAB (MathWorks, Cambridge, MA, United States). For each frame, the total motion power from 0 to 25 Hz (full motion spectrum) and the motion power from 8 to 12 Hz (the harmaline-induced tremor frequency bandwidth) were calculated. Next, the power spectra from 8 to 12 Hz was divided by the power spectra 0–8 Hz for each frame (Martin et al., 2005). Then, the resulting motion power ratio (MPR) for each 10-min epoch was generated by averaging the data from 1 frame. Each 10-min MPR was normalized by the averaged 20-min baseline MPR.

### Electrophysiological Recordings and Stimulation

Electrophysiological experiments were performed in anesthetized rats with urethane (1.5–1.6 g/kg, U2500-100G, Sigma-Aldrich, St. Louis, MO, United States). Eight tetrodes (nichrome wire) for recording were implanted into the M1 (AP + 2.7 mm, ML ± 3 mm from the bregma, and DV 0 to -2.4 mm from the brain surface) and the VLT (AP -2.3 mm, ML ± 1.8 mm from the bregma, and DV -5.6 mm from the brain surface). The tetrode is a bundle of four electrodes. A single-unit neuronal activity can be isolated from multi-unit signals by differential amplitude of action potentials over the four channels of tetrode (Nguyen et al., 2009). One concentric DBS electrode for stimulation was implanted in the VLT. Data were recorded with the Open Ephys platform^1^, an open-source data acquisition system based on Intan amplifier chips^2^ with or without electrical stimulation (130 Hz, 60 μs pulse width). Tetrode signals were band-pass filtered from 0.6 to 6 kHz, digitized at 30 kHz, and stored in a computer.

### Electrophysiology Data Analysis

All data analyses were performed using MATLAB. Units were clustered with MClust (David Redish, University of Minnesota). To demonstrate whether the firing rate during each period was changed, we calculated normalized index as a function of harmaline and DBS effects.

(1) Index=(B-A)/(B+A)

where *A* represents the number of spikes during the baseline state for 10 min and *B* indicates the number of spikes after harmaline injection and/or stimulation for 10 min. These were used to compare firing changes in neurons with different overall firing rates.

Neuronal firing patterns were classified into four categories: oscillatory-burst (os-burst), oscillatory-non-burst (os-non-burst), irregular-burst (irr-burst), and irregular-non-burst (irr-non-burst) (Supplementary Figure S1). Neuronal spikes for 10 min in each period were used to performed autocorrelation (ACH) followed by construction with a bin width of 1 ms (Bingmer et al., 2011). To minimize noise effect due to a low number of spikes, a Gaussian smoothing was applied with a 2-ms kernel, and then the central peak of the ACH was removed. Then, FFT was applied to calculate the power of frequency; and the threshold of power was determined by (mean of power) + 3*×* (standard deviation of power). If the power of neuronal spikes followed by FFT is over the threshold and within the range of 0.4 and 1.5 Hz, neurons meeting these criteria were considered as an oscillatory neuron. To classify burst cells, we defined burst as having a minimum of two spikes with a maximum interstimulus interval (ISI) of 10 ms and separated from other bursts by more than 100 ms (Fanselow et al., 2001). If the number of bursts was over 10% of the total number of spikes during each period, those cells were considered as a burst cell. With these two categories, four types of firing patterns were classified.

The coefficient of variation (CV) is a measure of spike train irregularity defined as the standard deviation divided by the mean ISI (Christodoulou and Bugmann, 2001).

### Histology

Upon completion of the experiments, the animals were deeply anesthetized with urethane (1,600 mg/kg) i.p. injection and perfused intracardically with saline followed by 10% formalin (Sigma Aldrich, St. Louis, MO, United States). Brain was extracted and immersed in 10% formalin solution overnight at room temperature. After fixation, brains were frozen, and 40 μm coronal sections were made using a microtome (Leica VT1000, Leica biosystems). The slices were stained by cresyl violet, and the positions of the electrode tips were identified on the basis of the atlas by Paxinos and Watson (1998).

### Statistical Tests

Statistical significance of each result was determined with a *t*-test. A *p* value < 0.05 was used as the criterion for a significant statistical difference. All data are expressed as mean ± SEM unless noted otherwise.

## Results

### Harmaline-Induced Tremor

**FIGURE 1:**
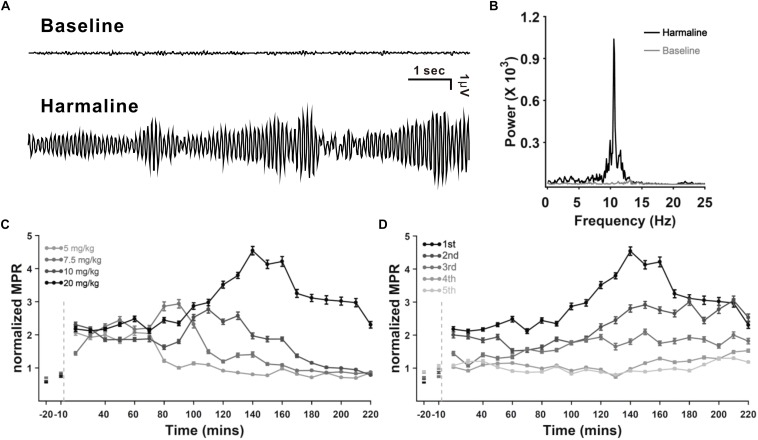
Harmaline-induced tremor in rats. **(A)** Example of raw tremor traces before and after harmaline injection (7.5 mg/kg). **(B)** Power spectra of frequency before and after harmaline injection, showing 8–12 Hz as the peak frequency of the harmaline-induced tremor. Tremor traces **(A)** were converted to power spectra of frequency by fast Fourier transform (FFT). **(C)** Dose dependency of harmaline effect. Motion power ratio (MPR) of tremor was calculated with a 10-min epoch. Harmaline-induced tremor was dependent on concentrations of harmaline (5, 7.5, 10, and 20 mg/kg). Light gray dashed line indicates the time point of harmaline injection. Error bars represent SEM. **(D)** Tolerance of harmaline effect. Repeated harmaline injection (20 mg/kg) reduced harmaline-induced tremor.

It is well known and evaluated that harmaline can induce tremor in a dose-dependent manner (Martin et al., 2005; Lee et al., 2014, 2018), and its repeated injection in rats leads to a desensitization of the tremorogenic effect of harmaline (Lutes et al., 1988). To evaluate thalamic DBS function in tremor, we used harmaline-induced tremor models with four doses of harmaline (5, 7.5, 10, and 20 mg/kg, i.p.) by administering each same dose consecutively for 5 days in each rat. Tremor amplitude, recorded by force plate actimeter (FPA), was significantly increased by harmaline injection (Figure 1A). Peak of motion power spectrum induced by harmaline was in the range of 8–12 Hz (Figure 1B). The power spectrum of frequency was transformed by the tremor amplitude as shown in Figure 1A. The time course of mean-normalized MPR of the harmaline-induced tremor is shown in Figure 1C. The overall averaged MPR of the first harmaline injection was dose dependent [mean MPR ± SEM, 1.20 ± 0.04, 1.6 ± 0.06, 1.8 ± 0.06, and 2.9 ± 0.1; 5, 7.5, 10, and 20 mg/kg of harmaline, respectively; *p* < 0.0001 by analysis of variance (ANOVA)]. Regarding tolerance of harmaline effect, our data were also consistent with those of a previous study (Lutes et al., 1988; Figure 1D), which showed a significant decline in mean MPR in comparison with five times of repetition. The mean MPR at total duration of 2.5 h after harmaline (20 mg/kg) administration from the first injection through the fifth injection was 2.9 ± 0.1, 2.3 ± 0.09, 1.7 ± 0.07, 1.1 ± 0.03, and 1.0 ± 0.03, respectively (*p* < 0.0001 by ANOVA). According to the maintained duration (data not shown) and tolerance of repeated harmaline injection, we decided to use 10 mg/kg of harmaline for our VL thalamic DBS function study.

### High-Frequency DBS Reduced Harmaline-Induced Tremor

**FIGURE 2:**
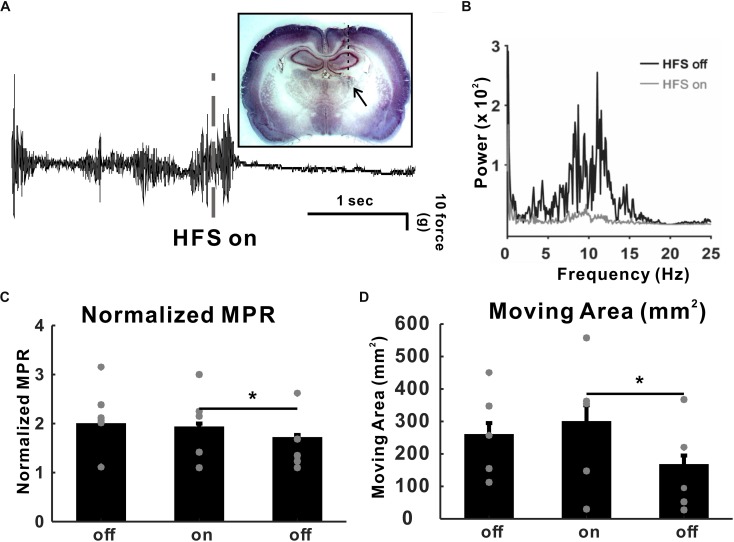
High-frequency deep brain stimulation (DBS) (HFS) effect in harmaline-induced tremor. **(A)** Raw tremor traces. Harmaline-induced tremor was reduced by electrical stimulation with 130 Hz (100 μs of pulse width, 50 μA intensity). The inset picture is the rat brain slice implanted with DBS electrode. **(B)** Power spectra of tremor before and during DBS. **(C)** DBS effect on harmaline-induced tremor. Average motion power ratio (MPR) for 10 min shows that harmaline-induced tremor was significantly reduced by HFS. The effect of electrical stimulation maintained for a while after HFS was terminated. Values are mean ± SEM. ^∗^*p* < 0.05 compared with each group using *t*-test. **(D)** DBS effects on mobility. Average moving area for 10 min was not significantly reduced by HFS, which indicates that MPR reduction during HFS-on period was not dependent on animal’s movement.

To determine the VL thalamic DBS effect, we implanted a DBS electrode unilaterally in the VLT (Figure 2A). We recorded tremor for 30 min immediately after harmaline injection, even though 30 min was not long enough for a full-tremor establishment by harmaline. However, considering that the onset time of tremor by 10 mg/kg of harmaline was 0.92 ± 0.24 min (data not shown), we found that tremor was reliably stable at 30 min post-harmaline injection. Once harmaline was injected, we recorded a tremor baseline for 5 min prior to DBS. Then, DBS was applied for 30 min and stopped for 30 min. This 30-min on–off DBS application was repeated twice. Tremor was significantly decreased by high-frequency DBS (130 Hz, 100 μs pulse width, 50 μA intensity) after 10 mg/kg of harmaline injection (Figure 2A). The power spectrum at 8–12 Hz was also significantly decreased by DBS (Figure 2B). The MPR was significantly reduced by DBS (Figure 2C). The next 30-min post-DBS MPR was also significantly decreased than the MPR in the DBS-on period. To determine whether those results could be affected by the animals’ movements, we calculated the gross moving area of the animal during each period. The animals’ movement during DBS-on period did not affect the MPR (Figure 2D).

### Harmaline Affected Neuronal Firing Rate in M1 and VLT

**FIGURE 3:**
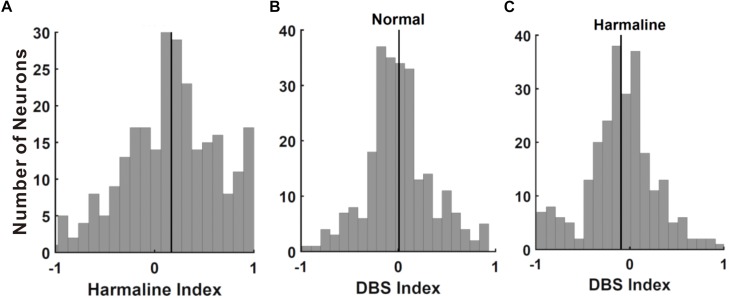
Effect of harmaline and deep brain stimulation (DBS) in the primary motor cortex (M1) neurons. **(A)** Distribution of cells by harmaline index. Black line indicates mean of M1 neuronal harmaline index, 0.154. Harmaline significantly increased the firing rates of 139 neurons and significantly decreased the firing rates of 67 neurons (*p* < 0.05, compared with the number of spikes for 10 min before and after harmaline injection (10 mg/kg) by Wilcoxon signed rank test). **(B)** Distribution of cells by DBS index. Black line indicates mean of M1 neuronal DBS index, 0.008. DBS significantly decreased the firing rates of 41 neurons and significantly increased the firing rates of 29 neurons (*p* < 0.05, compared with the number of spikes for 10 min pre-DBS and post-DBS by Wilcoxon signed rank test). **(C)** Distribution of cells by DBS index after harmaline injection. Black line indicates mean value of M1 neuronal DBS index, −0.094. DBS significantly decreased the firing rates of 119 neurons and significantly increased the firing rates of 65 neurons (*p* < 0.05, compared with the number of spikes for 10 min pre-DBS and post-DBS by Wilcoxon signed rank test).

To examine whether neuronal discharges in M1 and the VLT are changed by harmaline injection, we recorded neuronal activities in M1/VLT in anesthetized rats. A total of 369 neurons of M1 were recorded in eight rats, and 135 neurons of VLT were recorded in 15 rats. Following the previous literature, the recorded units in M1 were selected by putative pyramidal neurons (*n* = 338/369) on the basis of mean discharge rates (≤10 Hz) and spike width (<280 μs) (Barthó et al., 2004). Thalamic neurons were not separated into subgroups, because thalamic nuclei in rodents have less than 1% interneurons (Sherman and Guillery, 2001). Neurons having over 200 spikes during 20 min (10 min before and 10 min after harmaline injection) in both M1 (*n* = 249/338) and VLT (*n* = 116/135) were used for analysis, because neurons having less than 200 spikes in a 20-min period showed a tendency for neural signals to eventually fade away. First, we analyzed the harmaline effect on neuronal activities in the M1 and the VLT. Because harmaline-induced tremor appeared 1 min after harmaline injection, electrophysiology data were collected for 10 min after 1 min of harmaline injection to analyze the harmaline effect on the firing rate. According to the neuronal distribution in the harmaline index (Figure 3A), the background firing rate was significantly increased in the M1 (from 0.65 to 0.84 Hz in 159 cells out of 249 cells) and the VLT (from 0.68 to 0.86 in 79 cells out of 116 cells). Because each neuron fired differently, we calculated firing rate of each neuron as peak-normalized firing rate (firing rate of each block divided by max firing rate between before and after harmaline injection block). The ratio of firing rate increase is 0.17 ± 0.03 in the M1 and 0.14 ± 0.05 in the VLT (*p* < 0.0001 and *p* < 0.0001, respectively, by Wilcoxon signed rank test). Thus, the average firing rate increased consistently owing to harmaline injection in the harmaline index (Figure 3). The increase of the background firing rate was observed from 159 neurons in the M1 and 79 neurons in the VLT. On the other hand, the firing rates of 90 cells out of 249 cells in M1 and 37 cells out of 117 in VLT discharged were decreased after harmaline injection (*p* < 0.0001 and *p* < 0.0001 by Wilcoxon signed rank test, respectively).

### Four Different Firing Patterns of Neurons Changed by Harmaline

**FIGURE 4:**
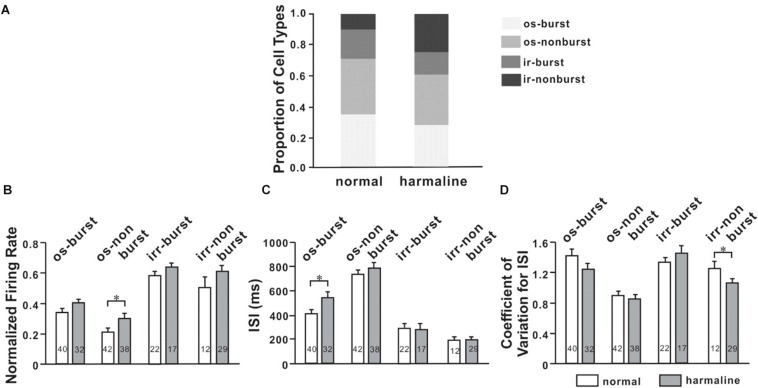
Altered neuronal activity patterns in the ventrolateral (VL) thalamus by harmaline. **(A)** Proportions of four different types of cells for each period (before and after harmaline administration). os burst, oscillatory-burst; os non-burst, oscillatory-non-burst; irr burst, irregular-burst; irr non-burst, irregular-non-burst. **(B)** Peak-normalized mean firing rate for each type of cell. The firing rate is significantly increased in the os-non-burst neuronal group only (*p* < 0.05 by Wilcoxon signed rank test). **(C)** Harmaline significantly increased the interstimulus interval (ISI) in only the os-burst group (*p* < 0.05). **(D)** Coefficient of variation for ISI is significantly decreased in only the irr-non-burst group (*p* < 0.05).

Oscillatory and bursting firing patterns in the cortex (Pasquereau and Turner, 2011; Lipski et al., 2017) and the thalamus (Fanselow et al., 2001; Fogerson and Huguenard, 2016) are thought to contribute to pathophysiology of movement disorders, like Parkinson’s disease (Pasquereau and Turner, 2011), and tremor (Swan et al., 2016). To evaluate this notion in our context, we recorded neuronal activities of M1 and VLT before and after harmaline injection and found various firing patterns in both M1 and VLT. To analyze discharge patterns, we used the burst-detection algorithm (described in the section “Materials and Methods”; Fanselow et al., 2001). We found that oscillatory neuronal proportion was significantly decreased (*p* < 0.0001; χ^2^-test) while burst neuronal proportion was not changed after harmaline injection in M1. We also performed the same analysis for VL thalamic neurons and found no changes in both oscillatory and burst neuronal proportions (Figure 4). In Figure 3A, the average harmaline index was positive, which means that a significant number of M1 neurons fired more after harmaline administration than before harmaline. Therefore, we examined which type of cells fired more after harmaline injection. In both areas, the firing rates of oscillatory-non-burst cells were only significantly increased (*p* < 0.0001; *p* < 0.05, M1 and VLT, respectively by *t*-test). These results indicate that the increased firing rate by harmaline injection might be driven by the increase of oscillatory-non-burst cells, which were the dominant population in the M1 and the VLT.

### DBS Reduced the Neuronal Firing Rate in M1 of Harmaline-Injected Animals

Local field potential (LFP) recordings of VIM show a strong linear correlation with the contralateral electromyography (EMG) during tremor (Marsden et al., 2000). Besides this, there are ample evidences suggesting the sensorimotor cortex is a part of the tremor-related oscillatory network with significant coupling between the M1 and the contralateral tremorogenic EMG (Hellwig et al., 2000, 2001; Govindan et al., 2006; Schnitzler et al., 2009; Muthuraman et al., 2012; Raethjen and Deuschl, 2012). Thus, we recorded M1 neuronal activities during high-frequency thalamic DBS in anesthetized rats before and after harmaline injection and then calculated the DBS index (Figures 3B,C). Most of the neurons were not affected by DBS in normal conditions (Figure 3B), whereas a significant number of neurons were changed by DBS in harmaline-injected conditions (Figure 3C; -0.09 ± 0.02, *n* = 249, *p* < 0.0001 by Wilcoxon signed rank test). The background firing rate was increased in 112 neurons among 249 cells from normal condition and in 98 neurons from harmaline condition.

### DBS Altered Neuronal Characteristics

**FIGURE 5:**
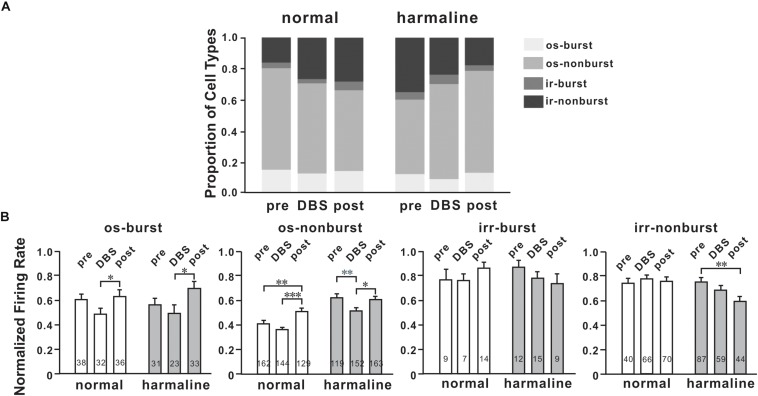
Characteristics of M1 neurons by deep brain stimulation (DBS). **(A)** Proportions of four types of cells for each period (before and after harmaline injection). os burst, oscillatory-burst; os non-burst, oscillatory-non-burst; irr burst, irregular-burst; irr non-burst, irregular-non-burst. **(B)** Peak-normalized mean firing rate for each type of cell. Each group of graphs explains four different types of neurons. White bars mean values before harmaline injection, and gray bars indicate values after harmaline injection. The numbers in the bars indicate the number of neurons for each group. ^∗^*p* < 0.05; ^∗∗^*p* < 0.01; ^∗∗∗^*p* < 0.0001 by Wilcoxon signed rank test.

To determine DBS effects on M1 neuronal firing patterns, we classified neurons into four firing patterns mentioned above. For the general population of M1 neurons, the proportion of oscillatory neurons was changed by DBS before (*p* < 0.05; χ^2^-test) and after harmaline injection (*p* < 0.05; χ^2^-test; Figure 5A). However, the number of burst neurons was not significantly changed by DBS before (*p* = 0.34; χ^2^-test) and after harmaline injection (*p* = 0.54; χ^2^-test). We also compared the proportion of burst neurons between pre-DBS and post-DBS after harmaline injection. Approximately 12.5% of M1 cells discharged in the oscillatory-burst pattern (os-burst), 47.8% of neurons discharged in the oscillatory-non-burst pattern (os-non-burst), 4.8% of neurons discharged in the irregular-burst pattern (irr-burst), and 34.9% of neurons discharged in the irregular-non-burst pattern (irr-non-burst). DBS altered the distribution of firing patterns of the M1 only in the os-non-burst and irr-non-burst groups (9.2% in os-burst, *p* = 0.25; 61.0% in os-non-burst, *p* < 0.01; 6.0% in irr-burst, *p* = 0.55; 23.7% in irr-non-burst, *p* < 0.01; χ^2^-test). Interestingly, even after the termination of DBS, the DBS effect was maintained (13.3% in os-burst, *p* = 0.79; 65.5% in os-non-burst, *p* < 0.0001; 3.6% in irr-burst, *p* = 0.5; 17.7% in irr-non-burst, *p* < 0.0001; χ^2^-test). Further analyses of each firing pattern using ISI and CV of it were performed, and the data revealed that ISI was not changed by DBS after harmaline injection. This means that the DBS effect is associated by altering distributions of neuronal firing patterns rather than changing in their own firing rate.

## Discussion

The aim of this study was to characterize DBS-associated neuronal changes in harmaline-induced tremor rats. Neuronal firing rates of the M1 were increased with harmaline administration, and this increased firing rate of the M1 was modulated by VL thalamic DBS. In addition, four firing patterns were identified in the M1 after harmaline injection; and the changes in the ratio of four firing patterns by VL thalamic DBS were characterized.

### Effects of VL Thalamic DBS in Harmaline-Induced Tremor Rats

A rodent model of harmaline-induced tremor has been used to unveil neural mechanisms of ET and test a new treatment for ET (Wilms et al., 1999; Deuschl and Elble, 2000; Martin et al., 2005; Miwa et al., 2006; Elble, 2014). Consistent with previous studies, we also showed harmaline-induced tremor in rats (Figure 1). Then, we found that the tremor amplitude was significantly reduced during VL thalamic DBS in harmaline-induced tremor rats; and the DBS effect remained for a while (Figure 2). To understand the underlying mechanisms of DBS at a single-cell level, we performed single-unit extracellular electrophysiology recording in anesthetized rats. The increased firing rates in M1 by harmaline were modulated by VL thalamic DBS, whereas there was no significant effect of DBS on M1 firing rates before harmaline injection (Figure 3). We also identified DBS effect on cortical firing patterns in a single-cell level in harmaline-treated rats. Previous studies have shown that DBS in the subthalamic nucleus (Welter et al., 2004) and internal globus pallidus (Dostrovsky et al., 2000) of humans showed decreased neuronal firing rates at the site of DBS during stimulation. In a more recent study, DBS at the motor thalamus has been shown to inhibit spike activity and modulate spike patterns locally using a single-unit electrophysiology measurement (Xiao et al., 2018). In this study, we also used a sophisticated single-unit electrophysiology and characterized the neural activity pattern at the M1 to determine DBS function distally. As most studies use LFP measurement at the M1 to investigate DBS mechanisms (de Hemptinne et al., 2015), our study may complement these previous studies by investigating electrophysiological changes before and after harmaline injection and before, during, and after DBS within the subject at the single-cell level.

### Specified Four Types of Neuronal Firing Patterns in M1

Specific firing patterns such as oscillation and burst have been regarded conveying information processing. It is known that visual thalamic theta oscillation is involved in sustained attention (Yu et al., 2018). Slow oscillation in cortical and thalamic networks is considered as a dynamic routing of information flow (Neske, 2016). In addition, thalamic bursting can be observed during periods of sensory processing (Fanselow et al., 2001). Thus, we classified four firing patterns of M1 and VL thalamic neurons as os-burst, os-non-burst, irr-burst, and irr-non-burst. A significant number of neurons having oscillatory patterns in both areas were decreased by harmaline, whereas numbers of burst neurons were not changed. We also examined the effect of DBS in firing patterns of M1 and found that the decreased number of oscillatory M1 neurons by harmaline reverted to what it was before harmaline injection by VL thalamic DBS (Figure 5). In addition, firing rates of oscillatory M1 neurons were decreased during DBS and returned to that of the pre-DBS period after terminating DBS. The DBS effect seemed to remain in proportion to firing patterns after termination of DBS (Figure 4A). In the post-DBS period, the proportion of cell types in M1 almost returned to that of the pre-DBS period even before harmaline administration. The same held true for tremor amplitude (Figure 1). Other DBS studies have shown that the DBS effect was present only during the DBS-on period (Kiss et al., 2002; de Hemptinne et al., 2015), which might be because short ranges of DBS were given. In our study, DBS was administered much longer than in other studies.

### Connectivity of M1 and VLT

Klein et al. (2012) showed that DBS in the ventralis intermedius (VIM) of the thalamus in humans – known as homologous with the VLT of rodents – has the common property of strong connections with the cerebellum and the VLT–motor cortex loop (Klein et al., 2012). The VLT has strong excitatory reciprocal connections with the motor cortex, premotor cortex, and posterior parietal cortex (Ilinsky and Kultas-Ilinsky, 2002), forming an excitatory loop that could amplify tremorogenic oscillations (Elble, 2013). There were similar neuronal firing patterns in VLT (Figure 4) and M1 (Figure 5) when we applied harmaline in rats, although we could not demonstrate a VL thalamic neuronal activity during DBS owing to a technical issue. The average firing rates of the neuronal population in both areas significantly increased after harmaline administration. In addition, a significant number of M1 neurons having an oscillatory firing pattern decreased (Figure 5A, *p* < 0.0001, χ^2^-test), whereas the number of VL thalamic neurons had a tendency to decrease, but not significantly (Figure 4A, *p* = 0.0948, χ^2^-test).

### Limitations of the Study

Our study has several limitations. First, we recorded neuronal activities of M1 and VLT under anesthesia. Urethane has been widely used for long-term anesthesia in laboratory rodents, because the magnitude of neuronal activity changes by urethane is less than that of other anesthetics such as ketamine and propofol (Hara and Harris, 2002). We could not fully rule out the urethane effect from our recording data; however, we could minimize the urethane effect by recording neuronal activities under the same anesthetic condition. In this study, we compared neuronal activities by harmaline and/or DBS under the anesthesia with urethane. Second, to test VL thalamic DBS for ET in rats, we currently used harmaline. A harmaline-induced tremor model is a well-established rodent tremor model, because its tremor frequency is comparable with ET in humans, clinical medication significantly reduces the tremor (Handforth, 2012), and cellular involvement of the inferior olivary nucleus has been identified (Llinas and Volkind, 1973). However, harmaline-induced tremor is an acute tremor, in contrast to chronic neurodegenerative ET in humans. Recent clinical data suggest neurodegenerative cerebella pathology in ET (Louis et al., 2007). It could be possible that various pathologic causes may generate ET in humans; thus, the pathology of a harmaline-induced tremor cannot fully mimic the pathology of ET. Finally, in our electrophysiology data, M1-oscillation frequency was in the range of 0.5–4 Hz, which is lower than our harmaline-induced tremor frequency. General anesthesia may decrease the oscillation frequency. However, we cannot rule out that this frequency discrepancy might be caused by a different type of signal transmission, because M1-controlled movement can be involved in sensory information processing to guide movement with accuracy (Heindorf et al., 2018). This low-frequency oscillation in M1 may convey sensory information. The reasons described above warrant further study of the DBS effect in awake tremor rodent models.

## Conclusion

The present study shows that the VL thalamic DBS modulated activity patterns of M1 neurons in harmaline-induced tremor rats. As an overall outcome, it is proposed that VL thalamic DBS can drive harmaline-induced M1 firing patterns back to normal patterns, which is suggested as a part of VL thalamic DBS mechanisms for tremor.

## Data Availability StateMent

The datasets generated for this study are available on request to the corresponding author.

## Ethics Statement

All experimental procedures were approved by the Mayo Clinic Institutional Animal Care and Use Committee for experimental animals.

## Author Contributions

JL and S-YC designed the study. JL did the experiments and performed the analysis. All experiments were performed at the Mayo Clinic, when JL was at the Mayo Clinic. JL and S-YC wrote the manuscript. Both authors approved the final manuscript.

## Conflict of Interest

The authors declare that the research was conducted in the absence of any commercial or financial relationships that could be construed as a potential conflict of interest.

## Footnotes

1 http://www.open-ephys.org

2 http://www.intantech.com
