# Supplementary figures and images for: Altered Primary Motor Cortex Neuronal Activity in a Rat Model of Harmaline-Induced Tremor During Thalamic Deep Brain Stimulation
Source: Front Cell Neurosci. 2019 Oct 15;13:448. doi: 10.3389/fncel.2019.00448 (PMC6803555; doi:10.3389/fncel.2019.00448)

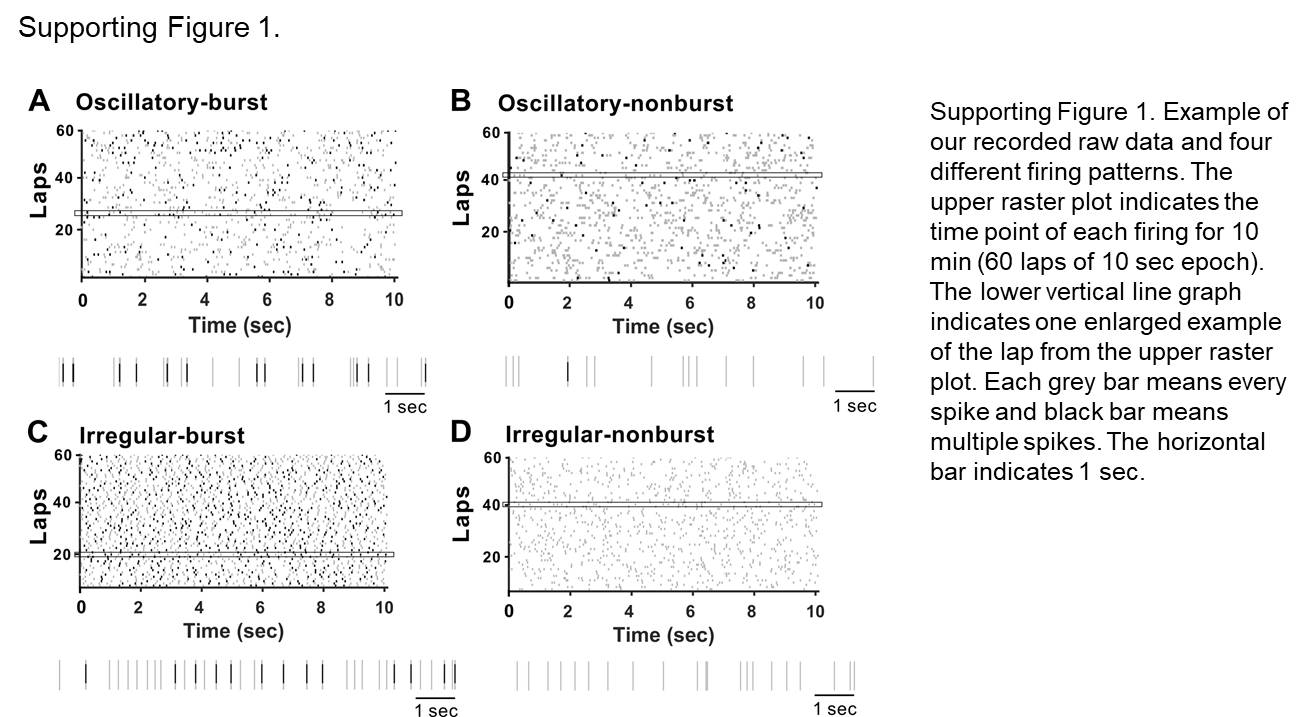

Supplement: Supplementary file 1 [file Image_1.jpg]
